# Gestational Diabetes Mellitus and Colostral Appetite-Regulating Adipokines

**DOI:** 10.3390/ijms25073853

**Published:** 2024-03-29

**Authors:** Jolanta Lis-Kuberka, Marta Berghausen-Mazur, Magdalena Orczyk-Pawiłowicz

**Affiliations:** 1 Division of Chemistry and Immunochemistry, Department of Biochemistry and Immunochemistry, Wroclaw Medical University, M. Skłodowskiej-Curie 48/50, 50-369 Wroclaw, Poland; magdalena.orczyk-pawilowicz@umw.edu.pl; 2 Department of Neonatology, J. Gromkowski Provincial Specialist Hospital, Koszarowa 5, 51-149 Wroclaw, Poland; 3 Faculty of Medicine, Wroclaw University of Science and Technology, Hoene-Wrońskiego 13c, 58-376 Wroclaw, Poland

**Keywords:** adipokines, gestational diabetes mellitus, human milk, lifestyle diseases

## Abstract

Gestational diabetes mellitus (GDM) is a complex metabolic disorder that has short- and long-term effects on maternal and offspring health. This study aimed to assess the impact of maternal hyperglycemia severity, classified as GDM-G1 (diet treatment) and GDM-G2 (insulin treatment) on colostral appetite-regulating molecules. Colostrum samples were collected from hyperglycemic (N = 30) and normoglycemic (N = 21) mothers, and the concentrations of milk hormones were determined by immunoenzymatic assay. A difference was found for milk ghrelin, but not for molecules such as adiponectin, leptin, resistin, or IGF-I levels, in relation to maternal hyperglycemia. The colostral ghrelin in the GDM-G1 cohort (0.21 ng/mL) was significantly lower than for GDM-G2 (0.38 ng/mL) and non-GDM groups (0.36 ng/mL). However, colostral resistin was higher, but not significantly, for GDM-G1 (13.33 ng/mL) and GDM-G2 (12.81 ng/mL) cohorts than for normoglycemic mothers (7.89 ng/mL). The lack of difference in relation to hyperglycemia for milk leptin, adiponectin, leptin–adiponectin ratio, resistin, and IGF-I levels might be the outcome of effective treatment of GDM during pregnancy. The shift between ghrelin and other appetite-regulating hormones might translate into altered ability to regulate energy balance, affecting offspring’s metabolic homeostasis.

## 1. Introduction

Adipokines are hormonally active molecules which play an important role in the regulation of biological processes in the human body. These cell-signaling proteins are released by adipose cells, establish intra-organ communication, and are considered the link between the endocrine and immune systems [1,2,3]. The anti-inflammatory adipokines, i.e., adiponectin and ghrelin, promote differentiation of M2 macrophages, are involved in anti-inflammatory responses, and attenuate differentiation of monocytes to pro-inflammatory M1 macrophages [4,5,6,7,8,9]. They are correlated negatively with BMI, insulin resistance, and body fat of diabetic persons [10,11,12,13]. Moreover, adiponectin is correlated negatively with fasting blood glucose, triglyceride, and waist circumference [12,13,14]. Pro-inflammatory adipokines, leptin, and resistin are positively associated with body weight, fat mass, insulin resistance, obesity, and type 2 diabetes.

Adiponectin and leptin are among the adipokines most often analyzed in maternal–infant dyads [15]. Hypoadiponectinemia and hyperleptinemia are involved in aggravation of insulin resistance and promotion of obesity-related low-grade inflammation, and are important biomarkers of metabolic disorders [16,17]. According to Bozkurt and coworkers [16], adiponectin and leptin plasma levels reflect deteriorated glucose metabolism at early gestation and may predict gestational diabetes mellitus (GDM). However, the leptin–adiponectin ratio (LAR) is a more accurate marker; for healthy individuals, the ratio is 1:2 [18,19]. An increased LAR value is an early indicator of adipose tissue dysfunction associated with an increase in insulin resistance, type 2 diabetes mellitus, and cardiovascular diseases. The imbalance between anti-inflammatory and pro-inflammatory adipokines contributes to vascular and endothelial dysfunction, immune cell infiltration, local and systemic inflammation [7,20], and manifestation of metabolic disorders [7,21,22]. So far, changes in adipokine pattern have been observed in plasma of individuals with obesity and diabetes, including GDM [7,8,9,23,24,25,26].

GDM is the most common complication of pregnancy, and its occurrence is an important issue for public health, since every year a significant increase is observed. Actually, approximately 15% of pregnancies are affected by GDM worldwide, which translates to ~18 million births annually [27,28]. During pregnancy, a woman’s body undergoes transformations, including accumulation of adipose tissue (30–50% weight gain). The rapid change in maternal body mass index may initiate the changes in adipokine release due to adipocytes or macrophages infiltrating adipose tissue, contributing to chronic low-grade inflammation, which are pivotal in the pathology of metabolic-related disorders [7,8,20,23,24,26]. Additionally, pregnancy predisposes to greater maternal insulin resistance, affecting the transport of glucose across the placenta and stimulation of fetal pancreatic insulin secretion [29,30,31,32,33,34]. These changes result in women having impaired glucose metabolism, which may translate into the development of gestational diabetes mellitus, associated with short- and long-term maternal and fetal adverse outcomes [35,36,37]. Mothers with ongoing hyperglycemia during pregnancy have a higher risk of occurrence of eclampsia, gestational hypertension, preterm delivery, and type 2 diabetes in comparison to mothers without hyperglycemia [35,36,37]. On the other hand, fetal exposure to maternal GDM increases the offspring’s risk of macrosomia, hypoglycemia, and adult-onset metabolic syndrome [38,39,40].

During pregnancy, molecules such as adipokines modulate nutrient transport through the placenta, which directly translates into fetus development [41,42,43,44]. After delivery, adipokines are transferred with maternal milk and they are crucial for postnatal development [15,44,45,46]. Milk leptin, adiponectin, ghrelin, resistin, and insulin-like growth factor-I (IGF-I) are appetite-regulating molecules involved in food intake regulation and energy balance in offspring [47,48,49,50,51]. Moreover, leptin, adiponectin, ghrelin, and resistin are involved in an infant’s growth trajectory [52,53,54,55,56]. The details concerning molecular processes by which maternal milk adipokines contribute to the growth and health of the neonates need to be clarified [57,58]. Milk leptin may act centrally to regulate food intake and also be able to exert effects on the gastrointestinal smooth muscle, thus helping in the creation of a satiety signal and ending breastfeeding [49,59,60,61,62]. In contrast, adiponectin stimulates food intake, participates in energy balance, and also is involved in lipid and glucose metabolism [49]. Resistin is an important link between obesity, insulin resistance, and diabetes [63,64,65] and its presence in human milk might contribute to regulation of an offspring’s growth, metabolic development, and appetite [57,66]. During the energy imbalance which occurs in pregnancy complicated by GDM, ghrelin participates in the adaptation of the developing fetus to the intrauterine environment [67]. After delivery, milk ghrelin might have an impact on regulation of gastric motility, acid, and insulin secretion [68]. In turn, the production of ghrelin and leptin is stimulated by insulin-like growth factor-I, which acts in concert with other appetite-regulating hormones and is important for the metabolism of healthy individuals [69,70]. IGF-I is structurally and metabolically similar to insulin but the exact role in appetite regulation and contribution to obesity and diabetes is still poorly understood [69,70]. Under normal conditions, IGF-I is more potent than insulin; however, in the plasma of obese patients, IGF-I was positively associated with body fat mass, while low IGF-I has been linked to obesity-related complications such as insulin resistance and type 2 diabetes mellitus [71,72]. In maternal milk, a higher concentration of IGF-I is associated with higher offspring weight at 13 months and lower weight at 3 and 5 years [73].

Currently, it is believed that the net result of altered adipokine levels in mothers may translate to the offspring’s development [15,49,51,62,66,74,75]. It was reported that appetite and satiety regulation are profoundly complex and involve the endocrine and the central nervous system [76,77]. The changes in appetite-regulating hormone pattern cause disturbances in the molecular signaling involved in control of food intake and feeling of satiety, which might translate into milk intake and possibly offspring growth [78,79,80,81]. The work groups [39,42] reported that the appetite-regulating molecules are a critical window for adiposity programming during the first 6 months of an infant’s life and predispose it to higher risk of obesity and metabolic disorders in later life. In light of the above, the aim of this study was to compare the concentrations of leptin, adiponectin, resistin, ghrelin, and IGF-I in colostrum samples collected from mothers with a hyperglycemic state of different severity levels, classified as GDM-G1 and GDM-G2, with a normal (non-GDM) group. Moreover, the correlations among appetite-regulating colostral molecules were analyzed. To the best of our knowledge, this is the first study that has examined colostral adipokines’ pattern.

## 2. Results

### 2.1. Characteristics of the Study Population

Fifty-one milk colostrum samples were provided by lactating gestational diabetic (N = 30) and healthy (normal glycemic) mothers (N = 21). Participant characteristics are outlined in Table 1.

The analyzed study cohorts did not differ in variables (the preconception BMI, maternal age, gestational age, and mode of delivery) that could affect the concentration of determined colostrum hormones. Overall, the maternal age was 33.38 ± 4.55 (median: 33) years and maternal preconception BMI was 24.16 ± 4.33 kg/m^2^, and these did not show significant differences among the analyzed groups of women with GDM-G1, GDM-G2, and non-GDM. More than three quarters of the analyzed cohort (80.39%) of mothers delivered newborns at term, namely at 38–41 weeks of gestation (GDM-G1: 75.00%, GDM-G2: 71.43%, and non-GDM: 90.48%), and the mothers who gave birth near to term (delivery at 36–37 weeks of gestation) constituted <30% of GDM cohorts and 9.52% of the non-GDM group (Table 1). In the analyzed cohort, the majority of pregnancies had been ended by cesarean section (74.51%) and in subgroups, the distribution of the analyzed factor was as follows: GDM-G1—75.00%, GDM-G2—78.57%, and non-GDM—71.43%.

The newborns’ birth weights did not differ significantly among the analyzed subgroups and averaged 3255.00 ± 583.57 g, 3172.14 ± 428.14 g, and 3434.29 ± 487.80 g, respectively, for GDM-G1, GDM-G2, and non-GDM (Table 1).

### 2.2. Concentrations of Adipokines in Colostrum Samples

**Figure 1:**
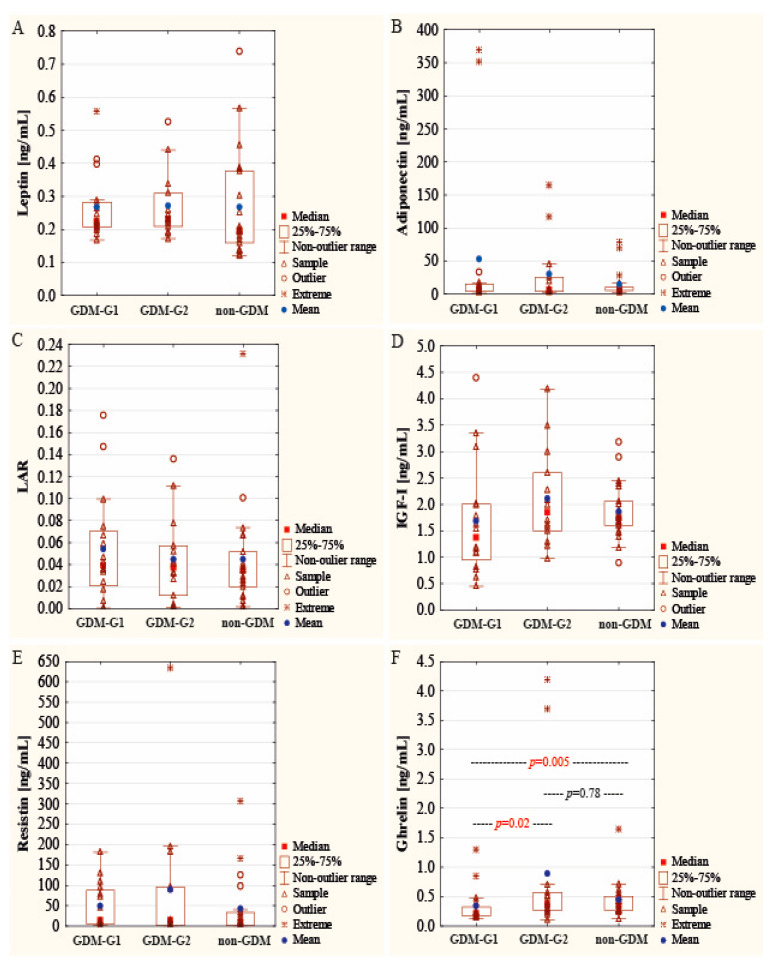
Comparison of leptin (**A**), adiponectin (**B**), LAR (**C**), IGF-I (**D**), resistin (**E**), and ghrelin (**F**) concentrations in colostrum between gestational diabetic (G1 and G2) and normoglycemic (non-GDM) mothers. Data are given as mean and median values and 25th and 75th quartiles. A *p*-value lower than 0.05 was regarded as significant.

The concentration of ghrelin in colostrum samples from the colostral period was significantly lower (0.21 (0.17–0.33) ng/mL) in the GDM-G1 group than in GDM-G2 (0.38 (0.27–0.57) ng/mL) and non-GDM (0.36 (0.27–0.51 ng/mL)) cohorts (*p* = 0.01) (Figure 1, Table 2).

The concentrations of other analyzed adipokines, namely leptin, adiponectin, resistin, and IGF-I were not significantly associated with maternal glycemic state. The median value of leptin was at a similar level namely 0.22 ng/mL (0.21–0.28 ng/mL), 0.23 ng/mL (0.21–0.31 ng/mL), and 0.20 ng/mL (0.16–0.38 ng/mL), for GDM-G1, GDM-G2, and non-GDM subgroups, respectively (Figure 1, Table 2). Similarly, the median value of adiponectin did not differ significantly in relation to maternal glycemic status and was 7.00 ng/mL (4.19–15.09 ng/mL), 6.44 ng/mL (3.94–25.80 ng/mL), and 6.84 ng/mL (5.45–10.57 ng/mL), for GDM-G1, GDM-G2, and non-GDM, respectively. Moreover, no significant differences for LAR were noted; it ranged from 0.035 to 0.039 in the analyzed cohorts. The median concentration of resistin in colostrum samples was 13.33 ng/mL (5.20–87.84 ng/mL) in GDM-G1, 12.81 ng/mL (1.13–96.11 ng/mL) in GDM-G2, and 7.89 ng/mL (1.82–33.14 ng/mL) in the non-GDM group (Figure 1, Table 2). The median IGF-I value for GDM-G1 was lower (1.36 (0.95–2.01) ng/mL), but not significantly, in comparison to GDM-G2 (1.84 (1.50–2.60) ng/mL) and non-GDM cohorts (1.74 (1.60–2.06) ng/mL).

### 2.3. Correlations of Adipokines and IGF-I with Maternal and Neonatal Outcomes

**Figure 2:**
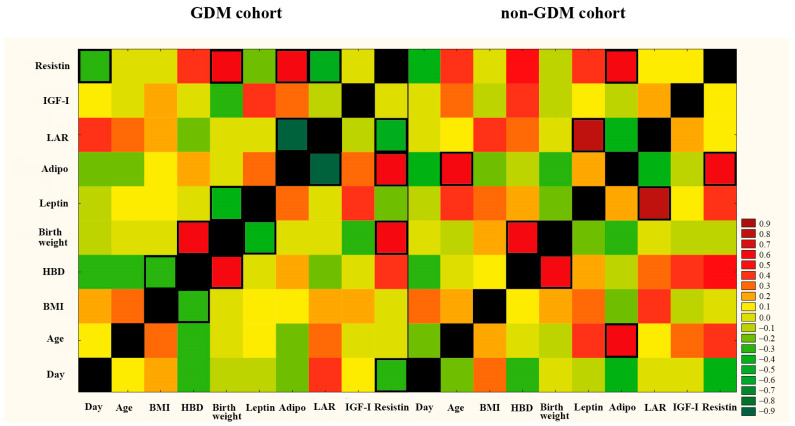
Correlations between concentration of adipokine in colostrum collected from GDM and non-GDM mothers and day of lactation, age, preconceptional BMI, and week of gestation. The Spearman correlation coefficient is represented in the heat map following the color in the legend. Adipo—adiponectin, BMI—preconceptional body mass index, LAR—leptin–adiponectin ratio, IGF-I—insulin-like growth factor-I; HBD—week of gestation. Bold frames represent correlations with statistical significance (*p* < 0.05).

To assess the influence of maternal age, preconception BMI, and the week of gestation on adipokines concentration in colostrum, the correlations between these parameters were studied (Figure 2, Supplementary Table S1). Due to the lack of significant differences (for leptin, adiponectin, LAR, resistin, and IGF-I) between cohorts of mothers with GDM-G1 and GDM-G2, these two subgroups were merged together.

In the non-GDM cohort, but not in the GDM group, a moderate positive correlation (r = 0.54, *p* < 0.05) was noted between adiponectin level and maternal age.

The concentration of adiponectin in colostrum from both cohorts, namely GDM and non-GDM groups, showed a positive correlation with resistin level (r = 0.58 and r = 0.53, *p* < 0.5, respectively) (Figure 2, Supplementary Table S1). Additionally, only for the GDM group was there observed a weak negative (r = −0.43, *p* < 0.05) correlation between leptin and neonatal birth weight and a positive (r = 0.50, *p* < 0.05) correlation for resistin level and neonatal birth weight. No correlation was found between IGF-I concentration and maternal anthropometric parameters (age, preconception BMI) in GDM and non-GDM subgroups (Figure 2, Supplementary Table S1).

#### Colostrum Ghrelin Reflects Maternal Hyperglycemia

To assess the relationship between maternal age, preconception BMI, the week of gestation, and neonatal birth weight with ghrelin concentration in colostrum, Spearman’s correlation values were analyzed (Table 3).

In the GDM-G1 group, a moderate negative correlation (r = −0.74, *p* < 0.05) was noted between ghrelin level and day of lactation. Additionally, only in this group was there observed a moderate positive correlation (r = 0.52, *p* < 0.05) for ghrelin and resistin concentrations.

In the GDM-G2 cohort, a moderate positive correlation (r = 0.62, *p* < 0.05) between ghrelin level and neonatal birth weight was observed. In contrast, for the non-GDM group, a weak negative correlation (r = −0.46, *p* < 0.05) was found for ghrelin and IGF-I concentration.

## 3. Discussion

The molecular processes underlying the pathology of GDM are not yet fully understood, and the impact of these changes on the developing fetus is even less known. Understanding the network of interconnections between maternal disease and health behaviors and their impact on child well-being is invaluable for the prevention of most diet-related metabolic disorders. Our study is the first to demonstrate the effects of maternal hyperglycemic states with different severity levels classified as GDM-G1 and GDM-G2 on the profile of the colostrum appetite molecules, namely leptin, adiponectin, leptin–adiponectin ratio (LAR), resistin, ghrelin, and IGF-I, which are important factors for newborn and infant growth.

Among analyzed colostrum hormones critical for adiposity programming, exposure to GDM translated into alterations in milk ghrelin. This study revealed significant differences between colostral ghrelin level and maternal hyperglycemic state. The ghrelin concentration in the GDM-G1 (0.21 ng/mL) cohort was significantly lower in comparison to the GDM-G2 group (0.38 ng/mL) as well as the non-GDM group (0.36 ng/mL). The observed lower ghrelin value for colostrum samples collected from mothers exposed to GDM classified as G1 (maintaining a proper glucose level by lifestyle modification with diet therapy), but not for GDM-G2 (insulin therapy), is the first such detailed comparison taking into account management of GDM. However, Aydin and coworkers [82] reported previously that the ghrelin level in milk from women with GDM is reduced, but they did not take into account the severity of maternal hyperglycemia. The observed differences in levels of ghrelin between analyzed cohorts, namely GDM-G1 and GDM-G2, might result from implementing insulin treatment and thus restore glucose level as in normoglycemic mothers [74,83]. In animal models, the high fat and fructose diet during pregnancy and lactation translate to higher ghrelin levels in rat’s plasma and milk [84,85].

In contrast, the maternal hyperglycemic state during pregnancy was not reflected in colostrum molecules, namely leptin, adiponectin, LAR, resistin, and IGF-I concentrations. The results for adiponectin are in line with data presented recently for colostrum by Mohamed and coworkers [86] and Luoto and coworkers [87]. On the other hand, the values for leptin and adiponectin (GDM-G1: 0.22 and 7.00 ng/mL, GDM-G2: 0.23 and 6.44 ng/mL, non-GDM: 0.20 and 6.84 ng/mL, respectively) are lower than data reported by Yu and coworkers [83] for the colostrum of GDM and healthy mothers. The lack of association between maternal hyperglycemic state and milk molecules, such as leptin and adiponectin levels, was reported previously [62,83,88]. Our data showed a weak negative relationship between colostrum leptin level and offspring weight, but for the GDM group only. The available data [86,89] indicate that adipokine concentrations might be related to milk maturation stages and, because of this, Çağiran Yilmaz and Özçelik [90] found a positive relationship between the first month milk leptin level and the mother’s anthropometric measurements (weight and BMI), but not with the newborn’s weight. Similar to our findings, other authors [91] did not find any associations between either milk leptin or adiponectin but found that maternal BMI was positively associated with milk leptin and insulin. Despite the fact that determinations of leptin and adiponectin levels in biological material are valuable, the leptin/adiponectin ratio has a higher diagnostic accuracy as a marker of insulin resistance and metabolic syndrome [92,93,94]. In our study, we did not observe differences in LAR values among GDM and non-GDM cohorts (GDM-G1: 0.039, GDM-G2: 0.037, non-GDM: 0.035), which is related to the lack of differences for colostrum adiponectin and leptin levels. So far, there has been one report concerning LAR values in maternal milk regardless of maternal glycemic status. Popova and coworkers [95] observed a significantly higher cord blood LAR value for the GDM-G2 group compared to non-GDM and GDM-G1 (GDM-G1: 0.97 ± 1.31, GDM-G2: 1.70 ± 1.66, non-GDM: 0.72 ± 0.46, *p* = 0.038), which was the result of a higher leptin level in GDM-G2, with a simultaneous constant level of adiponectin. It is worth emphasizing that milk leptin level was approximately 50 times lower than in cord plasma, which explains the differences in absolute values of LAR for milk and cord plasma. Moreover, significantly higher levels of diabetic individuals’ plasma leptin with parallel impaired adiponectin levels were also reported [96], although these changes were not reflected in maternal milk.

Although IGF-I levels were analyzed in relation to maternal glucose tolerance impairment during pregnancy [62,73,97,98], so far, no study has investigated their associations with GDM severity. Similarly to other researchers [73,98], we did not observe differences for IGF-I level between the analyzed cohort of GDM and non-GDM and also between colostrum IGF-I and maternal anthropometric parameters, such as BMI and age. The values for colostrum IGF-I for the non-GDM cohort (1.74 ng/mL) are in line with data presented previously [97] (2.7 ± 0.8 ng/mL). Although the analyzed periods of lactation are similar, the reported milk IGF-I level from the diabetic mothers [97] (44.97 ± 7.35 ng/mL) was higher than the values observed in this study (GDM-G1: 1.36 ng/mL, GDM-G2: 1.84 ng/mL). The observed values are the net result of differences in anthropometric parameters of offspring (neonatal weight, head and abdominal circumference), since higher IGF-I levels were found in plasma and cord plasma of GDM mothers [99,100] of macrosomic newborns. In light of the above, it can be speculated that these changes also translate into milk IGF-I level. Moreover, the compared cohorts differ in relation to week of gestation; namely, in this study, near-term birth constituted about one fourth of the GDM cohorts. Moreover, in contrast to the previous research [97], we did not observe a significant correlation between colostrum IGF-I level and infant weight in the GDM group, which is probably the result of the significantly higher newborn’s weight for analyzed groups, as discussed above.

The last of the colostral molecules evaluated in this study was resistin, an important player in diabetes and obesity. We found no significant differences in colostrum resistin level in relation to maternal hyperglycemic state (GDM-G1: 13.33 ng/mL, GDM-G2: 12.81 ng/mL, non-GDM: 7.89 ng/mL). However, these milk resistin values are higher than those reported by Ilcol and coworkers [101] and lower than data presented by Dalcin and coworkers [57]. In fact, we observed a higher, but not significantly, resistin level in relation to maternal hyperglycemia, while Dalcin and coworkers [57] noted the presence of significantly higher values for obese non-diabetes, normal weight diabetes, and obese diabetes groups in relation to the normal weight cohort. The data for resistin concentration in different stages of lactation (first and third months) reported previously [102,103] are lower than data obtained by us; this is in line with observations that resistin level decreases with progress of lactation and might be caused by the loss of fat stores in breastfeeding mothers [101,103]. So far, little is known about the relationships of resistin with other maternal milk adipokines. In this report, a moderate positive correlation was found between ghrelin and resistin levels, but only in the GDM-G1 cohort. For hyperglycemic mothers, significant associations between molecules such as resistin and adiponectin and between resistin and LAR values were found. On the other hand, for normoglycemic mothers, only a correlation between resistin and adiponectin concentration was revealed. Additionally, for the GDM cohort, a correlation between colostrum resistin level and birth weight of neonates was noted. Similarly to Santosa et al. [103], no associations between resistin and maternal anthropometric variables such as age and BMI were found.

The role of leptin, adiponectin, ghrelin, resistin, and IGF-I as appetite regulation molecules is well established, but there have been no general conclusions on their levels in human milk, especially in regard to the occurrence of maternal gestational diabetes [15,74]. The interpretation of the available results is hindered due to these gaps in knowledge, as well as lactation stage-related changes in milk, differences in geographic localization, varying sample collection time, and maternal health status (before and during pregnancy). In pregnancies complicated by GDM, diet and change in lifestyle (physical activity, and nutritional intervention) are often recommended to balance hyperglycemia [104]. The lack of difference in the pattern of leptin, adiponectin, LAR, resistin, and IGF-I levels between GDM and non-GDM mothers observed in our study might be the outcome of the implementation of quick and effective treatment of hyperglycemia during pregnancy. Moreover, the slight differences between previously reported levels of some milk adipokines are suggested to be related to different study populations in terms of lactation period as well as anthropometric characteristics of mothers and their offspring. Recently, Jara et al. [105] stated that even such a non-obvious factor as race affects adiponectin and leptin levels throughout pregnancy. Overall, appetite-regulating adipokines in human milk are suggested to affect newborn’s and infants’ milk intake and possibly offspring growth [91,106,107]. According to Adamska-Patruno and coworkers [108], the shift in the balance between serum ghrelin and other appetite-regulating hormones affects energy homeostasis [109,110,111]. Taking the above into consideration, it seems that the imbalance between colostral ghrelin and other appetite-regulating hormones might translate into altered offspring metabolic homeostasis; however, more investigations in this field are needed.

The indisputable strength of the present study is that a very homogeneous cohort of lactating women with respect to age, preconception BMI, week of gestation, and fetal growth pattern allowed the exclusion of potential factors that could have an impact on colostrum adipokine profile. However, limitations should be noted when interpreting the results. Firstly, in this study, we evaluated the appetite-regulating molecules patterns in colostrums with an emphasis on maternal glycemic state during pregnancy. Moreover, we focused on the analysis of selected colostrum hormone concentrations and relationships among them during the first week of lactation and we did not make comparisons to weight gain rates in later periods. Nevertheless, further analysis is needed, with larger, selected, and well-characterized study groups with additional assessment of anthropometric parameters of newborns and infants, which will make it possible to highlight potential variables that may affect the appetite-regulating hormone levels.

## 4. Materials and Methods

### 4.1. Recruitment of Breastfeeding Mothers

For the research, lactating, healthy, normoglycemic mothers (normoglycemic group, non-GDM group: N = 21) and women with GDM (hyperglycemic group, GDM group: N = 30), who received postpartum care in the First Department of Gynaecology and Obstetrics, located in Wroclaw Medical University (Wroclaw, Poland), were enrolled. The selection criteria of women with GDM were an abnormal fasting blood glucose level and/or oral glucose tolerance test (OGTT) following ingestion of 75 g of glucose [112,113,114,115].

The mother’s and infant’s health statuses were recorded and included the following variables: maternal age and preconception BMI, gestational age, mode of delivery, newborn’s birth weight, and newborn’s sex. The exclusion criteria included alcohol consumption and cigarette smoking during pregnancy, multiple pregnancy, delivery before 36 weeks of gestation, and birth weight not appropriate for gestational age.

The cohort of lactating mothers with gestational diabetes mellitus was divided into two subgroups: mothers with GDM controlled by diet (group GDM-G1) and with GDM controlled by diet and insulin (group GDM-G2) [112,113,114].

Overall, 51 women participated in the study and each mother donated only one milk sample. Specifically, the number of colostrum samples collected from healthy (normoglycemic, non-GDM) mothers was 21, from mothers with GDM-G1, it was 16, and from mothers with GDM-G2, it was 14.

### 4.2. Ethics

The study was approved by the Ethics Committee at Wrocław Medical University (No. KB-203/2022) and informed written consent was obtained from all participants. The study complied with the Declaration of Helsinki.

### 4.3. Colostrum Collection

Colostrum samples (1–7 days of lactation) were collected from a lactating woman at the First Department and Clinic of Gynaecology and Obstetrics, Wroclaw Medical University. Breastfeeding mothers, after breakfast, provided colostrum samples in a fixed period of time, between 08:00 and 12:00. Immediately after the collection procedure, the milk samples were frozen at −20 °C [115].

### 4.4. Colostrum Sample Pre-Treatment for Analysis

All collected colostrum samples were centrifuged at 3500× *g* at 4 °C for 35 min to remove fat and cells that interfere with the analysis. The obtained defatted colostrum samples were stored at −20 °C [115].

### 4.5. Determination of Adipokines and IGF-I Concentrations

The colostrum levels of adiponectin and resistin were measured using a commercial DuoSet kit (DY008 and DY1359, respectively; R&D Systems, Minneapolis, MN, USA) after previous adaptation of the assay for determination in milk samples. The detection ranges of the enzyme-linked immunosorbent assays (ELISA) were 15.625–1000 ng/mL and 31.25–2000 pg/mL, respectively. All defatted colostrum samples were assayed in duplicate. Colostrum samples were diluted (100 times for adiponectin and 20 times for resistin). The intra-assay and inter-assay coefficients of variation were 3.4% and 3.1% for adiponectin and 2.4% and 10.6% for resistin, respectively.

The colostrum levels of leptin and ghrelin were measured using a commercially available ELISA kit (DEE007; Demeditec Diagnostics GmbH, Kiel-Wellsee, Germany and E2142h, EIAab Science Co., Ltd., Wuhan, China) after previous adaptation of the assay for determination in milk samples. The defatted milk samples were transferred to plates without predilection. The detection ranges of the ELISA assays were 1–25 ng/mL and 0.156–10 and ng/mL, respectively. All defatted milk samples were assayed in duplicate. The sensitivity of the test was <0.25 ng/mL. The intra-assay and inter-assay coefficients of variation were 2.7% and 7.3% for leptin and 7.7% and 6.6% for ghrelin, respectively.

Colostrum levels of IGF-I were measured by an ELISA commercial kit (DEE020; Demeditec Diagnostics GmbH, Kiel-Wellsee, Germany) after previous adaptation of the assay for determination in milk samples. The detection range of the ELISA assays were 1 and 30 ng/mL, respectively. All samples were diluted 5 times and assayed in duplicate. The intra-assay and inter-assay coefficients of variation were 1.9% and 3.7%, respectively.

### 4.6. Statistical Analysis

The statistical analysis was done with TIBCO STATISTICA ver. 13.3 (StatSoft, Inc., Tulsa, OK, USA).

Categorical data such as the preconception BMI of mothers, type of delivery, and infant’s sex were presented as frequencies and percentages (% (n/N)). Continuous values were presented as the median, mean ± SD (standard deviation), and the twenty-fifth to seventy-fifth percentiles. The chi-square test was used to compare subgroups in the study population data. For analysis, nonparametric tests were used, since large interindividual differences are common in the biochemical profile of milk. The data were presented in the form of heat maps. Outlier analysis using the Tukey method showed the limits of typical observations. Values outside this range were reduced to limit values to reduce model load. The Kruskal–Wallis and Mann–Whitney U tests were used for the calculation of statistical significance. The correlations between analyzed groups were estimated according to Spearman. A two-tailed *p*-value lower than 0.05 was regarded as significant.

## 5. Conclusions

In recent years there has been increasing focus on characterization of the adipokine profile of the mother and/or newborn/infant and their potential impact on the early development of offspring. Colostrum adipokines transferred to newborn play an important role in regulation of metabolism, but their dysregulation is considered as a initiating factor of molecular processes that contribute to development of obesity-related dysfunction in later life. In light of the above, the evaluation of colostral appetite-regulating adipokines fill the gap in this field, since these molecules participate in nutritional programming of children. So far, most of the research has focused on one or two hormones, while our study showed the pattern of appetite-regulating adipokines in maternal colostrum in relation to maternal hyperglycemic status, with an emphasis on severity levels classified as GDM-G1 and GDM-G2. Except ghrelin, we did not observe significant differences in the concentration of colostrum hormones between analyzed cohorts. The lack of difference for colostral molecules, namely leptin, adiponectin, resistin, IGF-I, and leptin/adiponectin ratio might be the result of effectively managing GDM during pregnancy. Further studies are needed to evaluate milk hormone levels and relationships in successive lactation periods. Moreover, the potential impact of diet intervention on adipokine profile in maternal milk might be evaluated. Finally, the knowledge acquired in this area would potentially allow the improvement of artificial milk formulas to support optimized growth of newborns and infants.

Prevention of diabetes and obesity among the youngest members of society should be a priority, since the implemented actions are insufficient. The knowledge and understanding of correlations of maternal factors with colostral adipokines and translating these relationships into newborn’s health will be helpful in development and implementation of strategies in the prevention of obesity among children. The increasing evidence-based reports in this area and education of women of reproductive age might translate into conscious decision making concerning a natural way of feeding. Up to date, breastfeeding is considered the best documented nutritional intervention and has an undeniable impact on offspring development.

## Figures and Tables

**Table 1:** Characteristics of the study population.

	Overall *N* = 51 (% (n/N))	GDM-G1 *N* = 16 (% (n/N))	GDM-G2 *N* = 14 (% (n/N))	Non-GDM *N* = 21 (% (n/N))	Chi-Square Test χ^2^	*p*-Value
Race/Ethnicity White Europeans	100% (51/51)	100% (16/16)	100% (14/14)	100% (21/21)	-	-
Maternal age (mean ± SD)	33.38 ± 4.55	32.94 ± 4.68	34.79 ± 5.32	32.43 ± 3.63	4.55	0.60 (NS)
20–29	25.49% (13/51)	37.50% (6/16)	14.29% (2/14)	23.81% (5/21)		
30–34	33.33% (17/51)	31.25% (5/16)	28.57% (4/14)	38.10% (8/21)		
35–40	37.25% (19/51)	25.00% (4/16)	50.00% (7/14)	38.10% (8/21)		
40+	3.92% (2/51)	6.25% (1/16)	7.14% (1/14)	non		
Maternal pre-pregnancy BMI, kg/m^2^ (mean ± SD) Underweight (<18.5) Normal weight (18.5–24.9) Overweight (25.0–29.9) Obesity class 1 (30.0–34.9) Obesity class 2 (35.0–39.9)	24.16 ± 4.33 3.92% (2/51) 64.71% (33/51) 17.65% (9/51) 9.80% (5/51) 3.92% (2/51)	22.86 ± 4.12 6.25% (1/16) 68.75% (11/16) 18.75% (3/16) 6.25% (1/16) non	27.70 ± 5.62 non 35.71% (5/14) 35.71% (5/14) 14.29% (2/14) 14.29% (2/14)	21.90 ± 3.25 4.76% (1/21) 80.95% (17/21) 4.76% (1/21) 9.52% (2/21) non	13.88	0.08 (NS)
Gestational age (mean ± SD)	38.68 ± 1.35	38.75 ± 1.35	38.14 ± 1.12	39.14 ± 1.21	2.36	0.30 (NS)
Near term: 36–37 weeks	19.61% (10/51)	25.00% (4/16)	28.57% (4/14)	9.52% (2/21)		
Term: 38–41 weeks	80.39% (41/51)	75.00% (12/16)	71.43% (10/14)	90.48% (19/21)		
Delivery mode					0.31	0.85 (NS)
Vaginal birth	25.49% (13/51)	25.00% (4/16)	21.43% (3/14)	28.57% (6/21)		
Cesarean section	74.51% (38/51)	75.00% (14/16)	78.57% (11/14)	71.43% (15/21)		
Birth weight (g) (mean ± SD)	3287.14 ± 499.83	3255.00 ± 583.57	3172.14 ± 428.14	3434.29 ± 487.80	-	-
Appropriate for gestational age	100% (51/51)	100% (16/16)	100% (14/14)	100% (21/21)		
Newborn’s sex					4.53	0.33 (NS)
Male	49.02% (25/51)	62.50% (10/16)	57.14% (8/14)	33.33% (7/21)		
Female	47.06% (24/51)	31.25% (5/16)	42.86% (6/14)	61.90% (13/21)		
No information	3.92% (2/51)	6.25% (1/16)	non	4.77% (1/21)		

NS—not significant.

**Table 2:** Concentrations of appetite-regulating molecules in colostrum from diabetic mothers.

Colostral Molecules		Group			*p*-Value *	*p*-Value **		
	Overall N = 51	GDM-G1 N = 16	GDM-G2 N = 14	Non-GDM N = 21		G1 vs. G2	G1 vs. Non-GDM	G2 vs. Non-GDM
Leptin (ng/mL)	0.22	0.22	0.23	0.20	0.31	0.86	0.18	0.25
	0.19–0.31	0.21–0.28	0.21–0.31	0.16–0.38				
	(0.27 ± 0.13)	(0.27 ± 0.10)	(0.27 ± 0.10)	(0.27 ± 0.16)				
Adiponectin (ng/mL)	6.84	7.00	6.44	6.84	0.97	0.87	0.87	0.88
	4.68–16.12	4.19–15.09	3.94–25.80	5.45–10.57				
	(30.61 ± 73.63)	(52.58 ± 120.49)	(29.65 ± 49.39)	(14.50 ± 20.54)				
LAR	0.036	0.039	0.037	0.035	0.80	0.70	0.53	0.83
	0.017–0.066	0.021–0.070	0.012–0.057	0.020–0.052				
	(0.048 ± 0.047)	(0.054 ± 0.05)	(0.045 ± 0.04)	(0.044 ± 0.050)				
IGF-I (ng/mL)	1.70	1.36	1.84	1.74	0.21	0.13	0.15	0.73
	1.29–2.37	0.95–2.01	1.50–2.60	1.60–2.06				
	(1.88 ± 0.85)	(1.68 ± 1.09)	(2.11 ± 0.92)	(1.87 ± 0.54)				
Resistin (ng/mL)	11.98	13.33	12.81	7.89	0.55	0.79	0.27	0.61
	2.26–92.86	5.20–87.84	1.13–96.11	1.82–33.14				
	(56.73 ± 106.09)	(48.39 ± 56.41)	(89.46 ± 170.76)	(41.26 ± 75.77)				
Ghrelin (ng/mL)	0.30	0.21	0.38	0.36	0.01	0.02	0.005	0.78
	0.23–0.51	0.17–0.33	0.27–0.57	0.27–0.51				
	(0.53 ± 0.75)	(0.33 ± 0.31)	(0.88 ± 1.31)	(0.44 ± 0.31)				

* Kruskal–Wallis Test, ** Mann–Whitney U Test; Values are given as median, twenty-fifth–seventy-fifth percentiles and mean ± SD in parentheses. The Kruskal–Wallis and Mann–Whitney U test were used for statistical calculations, and a *p*-value lower than 0.05 was regarded as significant and were marked red color. LAR—leptin–adiponectin ratio.

**Table 3:** Correlation values between concentration of ghrelin and other adipokines and IGF-I in colostrum from GDM-G1, GDM-G2, and non-GDM mothers and day of lactation, age, preconceptional BMI, and week of gestation.

	Ghrelin [ng/mL]		
	GDM-G1	GDM-G2	non-GDM
Day of lactation	−0.74	−0.08	0.10
Age [years]	−0.14	0.52	0.21
BMI [kg/m^2^]	−0.16	0.11	−0.01
HBD [week]	0.28	−0.27	−0.28
Birth weight [g]	0.31	0.62	−0.12
Leptin [ng/mL]	−0.18	−0.42	0.12
Adiponectin [ng/mL]	0.33	0.11	0.36
LAR	−0.35	−0.16	−0.09
IGF-I [ng/mL]	−0.24	−0.40	−0.46
Resistin [ng/mL]	0.52	0.55	0.30

The correlations with statistical significance (*p* < 0.05) were marked red color. BMI—preconceptional body mass index, LAR—leptin–adiponectin ratio, IGF-I—insulin-like growth factor-I; HBD—week of gestation.

## Data Availability

The data underlying this article will be shared on reasonable request to the corresponding author.

## Supplementary Materials

The following supporting information can be downloaded at: https://www.mdpi.com/article/10.3390/ijms25073853/s1.
