# Supplementary material for: Gestational Diabetes Mellitus and Colostral Appetite-Regulating Adipokines
Source: Int J Mol Sci. 2024 Mar 29;25(7):3853. doi: 10.3390/ijms25073853 (PMC11011253; doi:10.3390/ijms25073853)
Supplement: Supplementary file 1 [file ijms-25-03853-s001.zip › ijms-2921936-supplementary.pdf]

Supplementary

Table 1. Correlations value between concentration of adipokine in milk collected from GDM and non-GDM mothers and day of lactation, age, preconceptional BMI and week of gestation.

	Day of lactation	Age [years]	BMI [kg/m^2]	HBD [week]	Birth weight [g]	Leptin [ng/mL]	Adiponectin [ng/mL]	LAR	IGF-I [ng/mL]	Resistin [ng/mL]
	**GDM group**									
**Day of lactation**	1.00									
**Age [years]**	0.09	1.00								
**BMI [kg/m^2]**	0.12	0.30	1.00							
**HBD [week]**	-0.32	-0.35	-0.37	1.00						
**Birth weight [g]**	-0.19	-0.01	-0.09	0.59	1.00					
**Leptin [ng/mL]**	-0.10	0.03	0.08	-0.03	-0.43	1.00				
**Adiponectin [ng/mL]**	-0.30	-0.29	0.01	0.14	-0.06	0.25	1.00			
**LAR**	0.31	0.27	0.10	-0.26	-0.10	-0.06	-0.96	1.00		
**IGF-I [ng/mL]**	0.01	-0.08	0.20	-0.02	-0.35	0.32	0.21	-0.16	1.00	
**Resistin [ng/mL]**	-0.39	-0.07	-0.04	0.32	0.50	-0.21	0.58	-0.55	-0.10	1.00
	**non-GDM group**									
**Day of lactation**	1.00									
**Age [years]**	-0.21	1.00								
**BMI [kg/m^2]**	0.23	0.16	1.00							
**HBD [week]**	-0.37	-0.06	0.05	1.00						
**Birth weight [g]**	-0.02	-0.16	0.15	0.55	1.00					
**Leptin [ng/mL]**	-0.18	0.36	0.26	0.12	-0.28	1.00				
**Adiponectin [ng/mL]**	-0.44	0.54	-0.27	-0.13	-0.39	0.15	1.00			
**LAR**	-0.09	0.01	0.30	0.23	-0.03	0.78	-0.41	1.00		
**IGF-I [ng/mL]**	-0.04	0.20	-0.14	0.30	-0.17	0.09	-0.14	0.11	1.00	
**Resistin [ng/mL]**	-0.45	0.37	-0.08	0.45	-0.18	0.34	0.53	0.08	0.08	1.00

The correlations with statistical significance ($p<0.05$) were marked red color.

BMI- preconceptional body mass index, LAR- Leptin/Adiponectin ratio, IGF-I- Insulin-like Growth Factor-I; HBD- week of gestation
